# When Is a Test Score Fair for the Individual Who Is Being Tested? Effects of Different Scoring Procedures across Multiple Attempts When Testing a Motor Skill Task

**DOI:** 10.3389/fpsyg.2017.00619

**Published:** 2017-04-25

**Authors:** Arve Vorland Pedersen, Håvard Lorås

**Affiliations:** Department of Neuromedicine and Movement Science, Faculty of Medicine and Health Sciences, Norwegian University of Science and TechnologyTrondheim, Norway

**Keywords:** assessment, performance

## Abstract

Tests or test batteries used for assessing motor skills, either in research studies or in clinical settings, apply a variety of procedures for scoring performances, including everything from one to ten attempts, of which the best is scored or an average is computed. The rationale behind scoring procedures is rarely stated, and it seems that the number of attempts allowed is decided without much qualification from research. It is uncertain whether procedures fairly capture an individual’s skill level. Thus, the validity of the tests may be compromised. The present study tested 24 young female soccer players on the juggling of a soccer ball. They were given 10 attempts, and trials were scored according to nine different procedures including the ‘best of’ or ‘mean of’ either one, two, three, five, or ten attempts. Individual raw scores differed widely across trials, but no general effect of trials was found. The mean (SD) percentage difference between the lowest and highest scores was 27.7(9.9)%, with 17 players (71%) demonstrating a significant change from lowest to highest score. Correlations between raw scores were low across trials, while they were generally higher across scoring procedures. The first trial was significantly different from the remaining both as a raw score and as scoring procedure. The mean percentage difference between best-of-two and best-of-ten scores was 95%, with 50 % of the players demonstrating a significant difference between the two scoring procedures. No significant differences were found across mean-of-rule scorings. Best-of-rule and mean-of-rule scorings were significantly different except for the best-of-two vs. mean-of-two. The mean difference between highest and lowest rank across players was 6.7 (3.6), with individual rankings within the group varying 33% on average across procedures. One player moved from 3rd to 23rd place because of procedural differences. Therefore, it is concluded that scoring procedures affect results and may have an impact on test outcomes. This may present consequences for decision-making from test results, such as diagnosing and selection of intervention groups. We hope that our results would inspire further research into the scoring procedures of the vast amount of tests and tasks in common use.

## Introduction

When assessing motor skills, whether in formal tests or in research studies, it is common to allow participants more than one attempt to ensure a fair result. The rationale behind such practice is seldom stated, but it is based on several relevant factors. Firstly, such a procedure reduces the risk of tested individuals achieving a poor result due to poor luck (or more scientifically termed, random fluctuations; Brown and Steyvers, 2009 or McManus and Ludka, 2012). Secondly, it increases the possibility of ensuring that the test result is representative of the skill that is being tested, thus increasing the validity of the test (Messick, 1980).

However, there is a limit to how many attempts are actually useful for a valid assessment of the underlying skill level. Therefore, time should not be spent on (extra) test attempts that do not provide relevant (extra) information. Furthermore, increasing the number of attempts, naturally, increases the time required for testing an individual, and consequently, the total time spent on testing. Time for test administration is a concern, especially within clinical settings (see Wiart and Darrah, 2001; Cools et al., 2009; Slater et al., 2010).

Sometimes, formal attempts are preceded by one or more practice or familiarization attempts, but the number of such attempts is generally restricted to avoid practice or learning effects (see Anastasi, 1985, 1986). Practice effects are perhaps most commonly discussed within cognitive tests (e.g., Collie et al., 2003; Bartels et al., 2010), but they are also a concern within psychomotor testing (Causby et al., 2014). Regardless of whether practice attempts are included, there does not seem to be much qualification regarding the choices of number of attempts across test-items, tests, and test batteries in current empirical data.

In studies of validity of motor skill testing, the focus has primarily been on convergent validity (Campbell and Fiske, 1959). Test scores from one test are often compared with scores from other tests that are already considered as valid (for example, see reviews by Wiart and Darrah, 2001; Deitz et al., 2007; Brown and Lalor, 2009; Cools et al., 2009; Slater et al., 2010). If the results from a new test match the old results or some other kind of “gold standard,” the new test is considered valid as well.

A less emphasized aspect of validity testing is the actual scoring of individual performances (which was not discussed in any of the above reviews). Test scores are often the focus of reliability testing, and it is seen as important to ensure that scores are similar across testers (inter-tester reliability) and across tests over time for each tester (intra-tester reliability) (Cronbach, 1947). Whether the testing procedures, and more specifically, whether the number of test trials/attempts are sufficient to capture the underlying skill-level of the individual who is being tested, has received less attention in research. Thus, test scores may be reliable, but they may not be particularly valid.

In one of relatively few studies to provide any form of qualification, Williams et al. (2009, p. 155) stated that their choice of number of trials (four) was based on “consultation with measurement specialists.”

Many tests or test batteries allow participants at least two formal trials (e.g., Ulrich, 1985; Henderson and Sugden, 1992; Henderson et al., 2007; Golle et al., 2015), often preceded by one or more familiarization trials after verbal instruction and demonstration of the task. Familiarization trials ensure that the individual understands the test item and the procedure, and may or may not be the exact, scored test-item. For example, the Movement Assessment Battery for Children, allows children a familiarization by means of a practice trial that is a shorter version of the scored trials on some items (Henderson and Sugden, 1992; Henderson et al., 2007). Other tests, however, include only demonstrations of the tested tasks together with explanations of the testing procedures (e.g., Williams et al., 2009).

How many attempts, then, are necessary to ensure that the underlying skill level has been captured fairly? It is obvious that allowing only one scored attempt carries a high risk of achieving a much poorer result than the individual’s actual skill level due to bad luck. Theoretically, it is also possible to obtain a result that is much better than the underlying skill level merely by chance but such luck is rarer. Should this happen, however, it is of equal importance to reduce the effect of such trials on the total score.

Allowing two attempts reduces the risk of ending up with an extremely poor result (a fail or near fail^1^) due to chance, and three reduces it even more. However, if the average score was recorded, such a ‘failed’ attempt would still count toward 50 and 33% of the score, respectively.

Therefore, most tests adopt of a reasonable compromise between the above-mentioned pros and cons, allowing participants at least two attempts, but seldom more than four, on a test item. Further scoring procedures, however, have been more diverse. Across standardized test batteries, scoring of performance varies across a wide range of procedures. These include using only the first attempt (the Peabody Developmental Motor Scales; see Wiepert and Mercer, 2002), counting the best out of two trials (the Movement Assessment Battery for Children, Henderson and Sugden, 1992; see also Van Waelvelde et al., 2004), computing the sum of two trials (the Test of Gross Motor Development; see Barnett et al., 2014), or scoring the average of three trials (the Purdue Pegboard Test; Tiffin and Asher, 1948). Furthermore, within the same test battery, one can also find item-specific scoring, with trials ranging from a single attempt up to seven attempts (Bruininks-Oseretsky Test of Motor Proficiency; see Deitz et al., 2007).

In their study, Wiepert and Mercer (2002) elegantly demonstrated that the number of test trials may be important for the outcome. Here, the authors showed that allowing for up to five trials on the Peabody Developmental Gross Motor Scale (the manual instructs testers to score the best out of two trials with minimal opportunity for practice) for 4- to 5-year-old preschool children resulted in a significant change in all gross motor skill domain scores. In comparison with the standard number of trials, the largest change occurred in up to three trials, with smaller additional changes in scores occurring with increases up to five trials. Wiepert and Mercer’s results revealed that almost 50% of the participants demonstrated clinically significant improvements in scores when multiple trials were allowed.

The present study included young female soccer players who were tested on the juggling of a football (soccer ball). They were allowed ten attempts when tested prior to a learning study from which the results will be reported elsewhere. Juggling a soccer ball is a commonly used test in research studies, and is also incorporated in test batteries, such as the F-MARC test battery for evaluating physical performance in football players (Rösch et al., 2000). This task has been scored in several manners across tests/studies, making it difficult to compare results. Vanderford et al. (2004) and Pedersen et al. (2014) counted the best out of two trials, while both Reilly et al. (2007) (best of two) and Vaeyens et al. (2006) (aggregate score from two) counted both attempts. Rösch et al. (2000) allowed three attempts, of which the best counted (in this study, players juggled with one foot).

Another aspect to consider when testing juggling, that will not be discussed further here, is the fact that most studies impose a ceiling, which limits the scores. This is done to limit the time spent on testing the skill, as many players can produce a fair amount of juggles, the occasional player several hundred. Among the mentioned studies, Rösch et al. (2000) counted a maximum of 25 juggles, while Vaeyens et al. (2006) counted up to 100. Vanderford et al. (2004) and Pedersen et al. (2014) set a slightly different limit, counting juggles within 30 s. Furthermore, an excessive number of juggles could introduce a decrement in performance due to fatigue but, as the task places relatively modest physical demands on a player, this is not likely not happen before the task has been ended for other reasons, such as lack of skill or by accident.

Based on the presented considerations, the principle aim of the present study was to investigate the effect of completing multiple trials on the same motor skill task (i.e., juggling a soccer ball) as well as the effect of different scoring procedures (‘best of’ versus ‘mean of’). It was hypothesized that there would be differences in scores across the various scoring procedures, and that these differences would affect players’ within-group rankings.

## Materials and Methods

### Participants

Twenty-four female soccer players participated in a motor skill learning study, from which the main results will be reported elsewhere. The participants belonged to the same under-16 soccer team, which was coached by the first author of the present study. The players were all 15–16 years old, and had 5–6 years of soccer experience. They were predominantly novel to the task of juggling a soccer ball, but all players had attempted the task, and some had a little experience with the task. The study was conducted in accordance with the Regional Ethics Committee for Medical Research and the tenets of the Declaration of Helsinki.

### Procedure

Players were tested one-by-one during one of their team’s training sessions. Hence, the players did not observe each other during testing and were unaware of the other players’ scores. All players performed 10 juggling trials in succession with a short break between trials, and no time limit was set for individual trials. No familiarization trials were given prior to the scored trials to secure that test conditions would be comparable with other studies not providing such trials. Players were tested outdoors on an artificial turf under similar weather conditions. All participants wore soccer cleats. Two training sessions spaced 1 day apart were sufficient to test all 24 players.

### The Task (Juggling)

Ball juggling is a test that is assumed to measure ball control, in which the frequency of consecutive and successful (i.e., preventing the ball from touching the ground) ball touches are counted, and higher values are deemed to represent a greater level of skill (Russel and Kingsley, 2011). In the juggling task for the present study, the players were instructed to keep the ball in the air without using their arms or hands, by means of various body parts. Thus, the task was to juggle the ball (regular soccer ball, size 5) as many times as possible, where the score was the number of hits on the ball before it fell to the ground. Counting stopped when the ball hit the floor, and no time limit or any other limitation that would induce a ceiling were set. The players were informed that if the same body part was used two times (or more) in succession, it was counted as one juggle (as in Pedersen et al., 2014). This ‘consecutive touch’ rule was applied to avoid excessive use of repeated preferred foot wrist juggling (a far easier task). Furthermore, the task was taken from The Football Association of Norway [NFF] (2016) standardized test of technical skills in children, for which the instructions include the ‘consecutive touch’ rule.

### Scoring Procedure

To investigate the effect of the number of trials on the assessment of individual performance on the juggling task, the performance on the first trial was identified and a *‘best of rule’* or *‘mean of rule’* was applied across two, three, five, and ten trials.

### Statistical Analysis

Non-parametric statistics were applied due to a modest sample size, i.e., one could not expect data to be normally distributed or that the sample was drawn from a population with normally distributed scores on the juggling task. Thus, Friedman’s test was applied to assess the effect of trials on raw scores and scoring procedures upon the ranking of players, with Kendall’s coefficient of concordance as a measure of effect size. *Post hoc* analysis was conducted with Wilcoxon tests. The relationship between different methods of scoring performance across trials, as well as the relationship between raw scores from the ten trials, was examined with Spearman’s *rho* correlations. To further examine the occurrence of significant changes in scores from different trials and across scoring procedures, a significant change was operationally defined as scoring outside the 95% confidence intervals of the mean change in measurements (Bland and Altman, 1986; Wiepert and Mercer, 2002). The statistical significance level criterion was set at *p* < 0.05.

## Results

**FIGURE 1:**
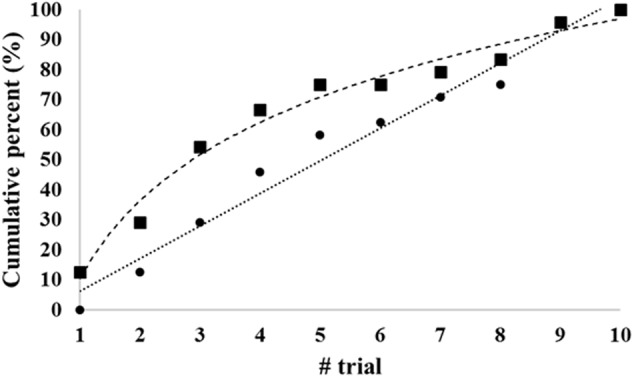
**Trial numbers at which players, on average, reached scores of 90% (▪) and 100% (●) of their ‘best-of-ten’ scores**.

Descriptive statistics for the raw juggling scores can be found in **Table 1**. Overall, there was no significant effect of trials on the raw scores (Friedman test: χ*^2^* = 13, *df* = 9, *p* > 0.05; Kendall’s *W* = 0.06). *Post hoc* tests, however, indicated significant differences between performance on the first trial and the other trials (*Z* > 2, *p* < 0.05). As depicted in **Table 2**, low or no statistical significant correlation coefficients were also found between scores from the different trials. Players’ lowest scores occurred, on average, on the sixth trial (SD: 3.14), while their highest scores, similarly, occurred on the fifth trial [mean (SD): 5.5 (2.7)]. This pattern of results is also reflected in **Figure 1**, which shows that most players required multiple attempts to reach their best score on the task. Additional analysis of the raw scores indicated that the mean (SD) percentage difference between the lowest and highest scores was 27.7(9.9)%, with 17 players (71%) demonstrating a significant change from lowest to highest score outside the 95% confidence interval (CI) (Low: 5.4, High: 9.8).

**Table 1:** Descriptive statistics for raw scores (*n* juggles) in the juggling task (*n* = 24).

Trial#	Min	Max	Mean
1	1	10	3.42
2	1	20	5.38
3	2	13	5.42
4	1	27	5.42
5	1	16	5.04
6	2	11	4.79
7	1	13	4.54
8	2	13	5.33
9	1	13	5.21
10	1	20	5.58

**Table 2:** Intercorrelations (Spearman’s *rho*) between raw scores across 10 trials (*n* = 24).

Trial#	1	2	3	4	5	6	7	8	9	10
1	1	0.35	0.28	0.31	0.30	0.18	**0.64**	0.23	0.14	0.27
2		1	**0.53**	0.38	**0.76**	**0.43**	**0.44**	0.25	0.29	**0.48**
3			1	0.28	**0.45**	0.39	0.25	0.19	0.40	0.20
4				1	0.32	**0.43**	**0.44**	0.35	0.27	**0.52**
5					1	**0.49**	**0.51**	0.31	**0.45**	**0.60**
6						1	0.35	0.19	0.19	0.25
7							1	0.21	0.22	**0.63**
8								1	0.16	**0.46**
9									1	**0.51**
10										1

*Significant correlation coefficients (*p* < 0.05) in bold.*

**FIGURE 2:**
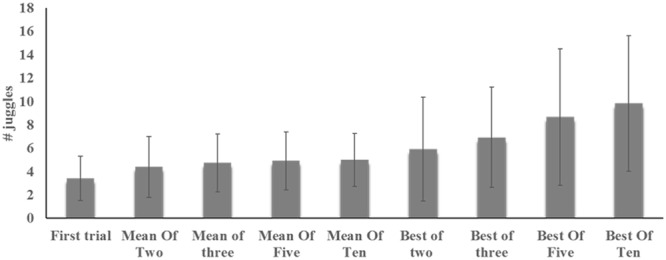
**Mean (SD) juggling performance across participants as assessed by first trial and across ‘best of rule’ or ‘mean of rule’ scoring procedures**.

**FIGURE 3:**
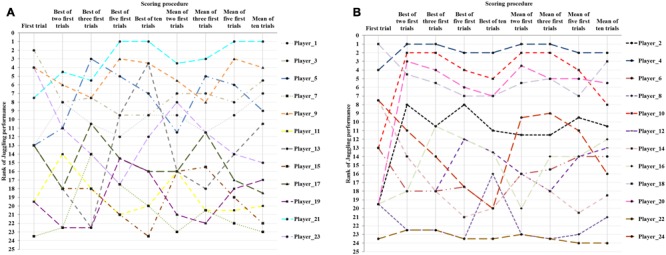
**Individual rankings (1–24) in the sample according to scoring procedure**. The figure is split in two for better visibility.

Performance on the juggling task across different scoring methods can be found in **Figure 2**. As evident from the figure, the first trial generated the lowest performance [mean (SD): 3.4 (1.9)] with an increase up to best of ten trials [mean (SD): 9.8 (5.8)]. The mean-of-trials scoring all amounted to similar scores with mean (SD) ranging from 4.4 (2.6) up to 5.0 (2.3) juggles. As depicted in **Figure 3**, multiple trials and different scoring procedures introduced considerable fluctuations in the players’ ranking within the group. The mean (SD) of difference in highest and lowest rank across players was 6.7 (3.6). As an example of the latter ranking effect, an individual player in the sample was ranked third in one scoring procedure and 23rd in another scoring procedure.

There was an overall significant effect of scoring procedure on juggling performance (Friedman test: *χ^2^* = 126, *df* = 8, *p* < 0.001; Kendall’s *W* = 0.66). *Post hoc* analysis indicated that performance on the first trial was significantly different from all other forms of scoring (*Z* > 2, *p* < 0.05). The ‘best of’ rule introduced statistically significant increases in performance scores when players were given multiple trials (Friedman test: *χ^2^* = 47, *df* = 3, *p* < 0.001; Kendall’s *W* = 0.66). Follow-up with Wilcoxon tests indicated significant differences across all best-of-rule scorings (*Z* > 2.6, *p* < 0.01). Further analysis regarding change in scores according to the ‘best-of-rule’ indicated a mean percentage difference between best-of-two and best-of-ten scores at 95%, with 12 players (50%) demonstrating a significant change between the two scoring procedures outside the 95% CI (Low: 2.2, High: 5.6).

By contrast, there were no significant differences across mean-of-rule scorings (Friedman test: *χ^2^* = 7.1, *df* = 3, *p* > 0.05; Kendall’s *W* = 0.1). In direct comparison between best-of-rule and mean-of-rule scorings, however, all were significantly different (*Z* > 2.9, *p* < 0.01), except for the best-of-two scorings that were not significantly different compared to the mean-of-rule scorings (*Z* < 2.3, *p* > 0.05). Further analysis indicated that the mean percentage difference between best-of-ten and mean-of-ten scoring procedures were at 55%, with 15 players (63 %) demonstrating a significant change between the two scoring procedures outside the 95% CI (Low: 3.3, High: 6.4).

As presented in **Table 3**, there were significant intercorrelations between all scoring procedures, with correlation coefficients ranging from 0.40 to 0.66 for performance on the first trial vs. all other conditions, 0.69 to 0.92 within best-of-rule scorings, 0.83 to 0.95 within mean-of-rule scorings, and intercorrelations ranging from 0.73 to 0.97 for best-of-rule vs. mean-of-rule scoring procedures.

**Table 3:** Intercorrelations (Spearman’s *rho*) between different scoring procedures.

	1	2	3	4	5	6	7	8	9
(1) First trial	1	0.59	0.40	0.41	0.45	0.66	0.59	0.55	0.58
(2) Best of two first trials		1	0.84	0.78	0.74	0.97	0.90	0.90	0.85
(3) Best of three first trials			1	0.81	0.69	0.82	0.94	0.86	0.77
(4) Best of five first trials				1	0.92	0.78	0.83	0.93	0.92
(5) Best of ten trials					1	0.74	0.73	0.86	0.91
(6) Mean of two first trials						1	0.93	0.91	0.84
(7) Mean of three first trials							1	0.93	0.83
(8) Mean of five first trials								1	0.95
(9) Mean of ten trials									1

*All correlation coefficients are significant (*p* < 0.05).*

## Discussion

The aim of the present study was to investigate the effects of varying the scoring procedures on test scores and individual rankings within a group of young female soccer players tested on juggling a soccer ball. Players were given 10 trials, which were all scored, and differences across nine different scoring procedures were analyzed.

The averaged results for the participants increased with the increasing number of attempts when applying the ‘best of’ rule (**Figure 2**). The increase was almost linear, and occurred because the best results from early attempts would be included in later scoring procedures. Thus, individuals can only change their best result by improving it and the average best result can never become worse. When scoring the performances using the ‘mean of’ rule, the results did not improve similarly. Here, the effect of poor attempts is stronger, with each poor attempt counting as 1/10 of ten trials, 1/5 of five trials, and so on. The largest effect, obviously, comes when scoring only the first attempt, as a poor result here would be extremely unfavorable and might not at all reflect the underlying skill level. Also, for other procedures involving scoring by the ‘mean of’ rule, the results are far below those for scores involving the ‘best of’ rule. There was a significant difference in average raw scores between the first trial and each of the remaining trials, but no other differences across trials. Correlations between trials were generally low or non-significant, indicating large variations across trials. The average score on the first trial was low compared with all other trials, but especially compared with the last trial, for which results were on average 63% better than those for the first trial. When comparing across scoring procedures, scoring the first trial again came out as different from all other scoring procedures, indicating that this procedure has a great risk of producing test-results that would be unfair toward individuals (here: players) as they are far below the players’ potentials.

The present study did not provide the players with any familiarization trials. The reason for this decision was that many tests or test batteries included no familiarization trials (for example, neither Wiepert and Mercer, 2002, nor Williams et al., 2009 included such familiarization), and we would not be able to compare our results with those trials if familiarization trials were given. However, on inspection of the present results, it is argued that the first trial should not be scored as the test result, or, at least, familiarization trials should be given first. However, as is evident from the increase in scores when applying the ‘best-of’ rule, the second trial also fails to capture the potential of the players.

What is even more interesting than the increase in average performance with more trials is the fact that such linear increases were not evident in individual series of trials. Players produced their best result on any attempt between the second and the tenth, and there was no clear trend, as is also indicated by the lack of increase in average (‘mean of’) scores, mentioned above. One player produced her best result (20 juggles) on her second attempt, and never eclipsed that performance in later trials. On the other hand, one player did not reach her maximal potential until the fourth attempt, when she completed 27 juggles, a result that still stood as her best after 10 trials (although she did juggle 20 on her 10th trial). On average, the players produced their best result on their fifth trial. Additionally, when inspecting relatively poor trials, the picture is similar. The poorest attempt for each individual player occurred anywhere from the first throughout the last attempt. On average, players produced their poorest result on their sixth attempt, but no trend was found to exist for poor scores.

Correlations of results across scoring procedures ranged between 0.40 (first trial and best of three) and 0.97 (best of two and mean of two) (**Table 3**). For the ‘mean of’ rule, correlations were generally higher than for the ‘best of’ rule (0.83 to 0.95 vs. 0.69 to 0.92, respectively), while ‘best of’ and ‘mean of’ for the same number of attempts also correlated strongly (0.91–0.97). This might lead to the conclusion that it does not matter which procedure is chosen, particularly if we avoid using the first trial as the scored one. However, we cannot use these results to determine how many trials should be allowed unless we can find some kind of ‘gold standard’ to correlate them with. The present study does not include any such ‘gold standard,’ but the argument could be made that in either procedure involving all ten trials (‘best of’ or ‘mean of’), the effect of very poor trials due to poor luck would be relatively smaller. The main argument against using as many as 10 trials is the time factor, and it seems that a procedure including five trials might be an acceptable compromise, at least in the present dataset, as the ‘best-of-five’ procedure came out as strongly correlated with ‘best-of-ten’ (0.92), and also produced an average result for the players of 8.7 juggles as compared with 9.7 for ‘best-of-ten’. The mean-of-five and the mean-of-ten correlations are also high (0.97), and results for the two procedures are similar, so it could be argued that as many as ten trials are perhaps not necessary to ensure fair test results. In fact, as many as 75% of the players had produced a result within the first five attempts that was close to (> 90%) their maximum for ten attempts, a number that did not change much until the ninth and tenth attempts (see **Figure 1**). Furthermore, nearly 60% of the players had, in fact, produced their best result of the ten within the first five trials.

Applying the ‘mean of’ rule to individual players’ raw scores resulted in scores of 51–74% of their ‘best of’ scores for an equal number of attempts. This indicates that players, on average, may not be able to produce results that are close to their potential, which may, as mentioned earlier, be due to an unduly large effect of poor scores. Furthermore, one of the characteristics of lower skill levels (as in the present study), is the lack of ability to reproduce performance across trials.

The entire picture becomes even messier when players are ranked according to their results across scoring procedures. **Figure 3** shows how players’ rankings within the group change across scoring procedures, and the reader is reminded that in an ideal world (in which scoring procedures did not matter), we would see 24 horizontal lines. In fact, individual players’ rankings changed, on average, eight places (33%) across scoring procedures.

The largest drop in ranking occurred for player 13, who was ranked third out of 24 when scoring results after the ‘best-of-ten procedure,’ while she was only number 23 out of 24 on the ‘best-of-three procedure’. Player number 21 stood out on all measures from five trials upward. She was, however, not ranked on top on either of the measures involving fewer than five trials, thus her skill level relative to the remainder of the players would not be captured by most of the commonly applied testing procedures mentioned earlier. The mean percentage difference between players’ lowest and highest scores was 27.7 (9.9%), and for as many as 17 of the players (71%), the difference was significant, falling outside the 95% CI. Thus, differences across scoring procedures in the present study showed a similar picture to the clinically significant improvements of children reported by Wiepert and Mercer (2002).

We would be extremely careful in recommending changes to any existing testing procedures based on this relatively modest experiment, but would offer a few thoughts based on our findings. The generalizability, of course, will vary across tests and tasks, perhaps particularly across tasks of different complexity and degrees of difficulty (Fitts, 1954; Joseph et al., 2013). However, it can be argued that it is fairer to the participants (here: players) to use a ‘best of’ rule, at least when scoring relatively few test attempts, compared with a ‘mean of’ rule, as the latter would place undue weight on poor attempts (which may occur out of pure mishap). If one should use a ‘mean of’ rule, the mean should include at least five attempts to reduce unproportionally large effects of (bad) luck. This seems like an acceptable compromise when considering time spent for testing, as the best of ten and best of five did not differ more than 12%, and results for these two procedures also correlated highly. For example, it seems very unfair for a player who can produce 27 juggles (specifically, player 21 in the present sample) to be assigned a poor result based on the ‘first trial’ that was four juggles, or seven juggles, which was the ‘mean-of-two.’ Such considerations, however, must be evaluated against the specific requirements of the task, and repeated testing may be required until an asymptote of performance has been reached in order to establish an exact number of necessary trials.

The results have consequences for any testing of motor skills, as well as for (neuro-) psychological tests, or test batteries, and a plethora of tests used in clinical studies. More specifically, the consequences of applying testing procedures that are unfair to participants in a screening might inflate the numbers of individuals who are being diagnosed with certain diseases (Wiepert and Mercer, 2002) or selected for groups that receive various interventions, as results of motor skill tests are often included as part of the assessment. Furthermore, when used in pre-tests for intervention studies, unfair testing procedures might induce effects of interventions that are not accurate. In fact, if the departure point (pre-test) is poor enough, which could simply be due to (bad) luck, almost any intervention may come out as effective.

## Conclusion and Limitations

The present study included data from a mere 24 participants, on only one task. It is not certain whether the results would be reproducible for other types of tasks. Still, the present study produced highly significant results from its modest quantity of data. It is reasonable to assume that the inclusion of more participants would increase the variability and thereby strengthen the findings. Generalizability across tasks, however, is a matter that should be further studied. It is not possible from the present results to argue which of the scoring procedures is the best, but rather to point to the large differences across procedures and encourage researchers to further explore this effect.

## Author Contributions

Study conception and design: AP and HL; Acquisition of data: AP; Analysis and interpretation of data: AP and HL; Drafting of manuscript: AP and HL; Critical revision: AP and HL.

## Conflict of Interest Statement

The authors declare that the research was conducted in the absence of any commercial or financial relationships that could be construed as a potential conflict of interest.

## Footnotes

1 Many tests, in fact, allow a new trial instead of scoring failed attempts.
